# Supplementary material for: Mechanistic insight into the role of AUXIN RESISTANCE4 in trafficking of AUXIN1 and LIKE AUX1-2
Source: Plant Physiol. 2023 Sep 30;194(1):422–33. doi: 10.1093/plphys/kiad506 (PMC10756756; doi:10.1093/plphys/kiad506)
Supplement: kiad506_Supplementary_Data [file kiad506_supplementary_data.zip › Tidy et alSupplemental Data.pdf]

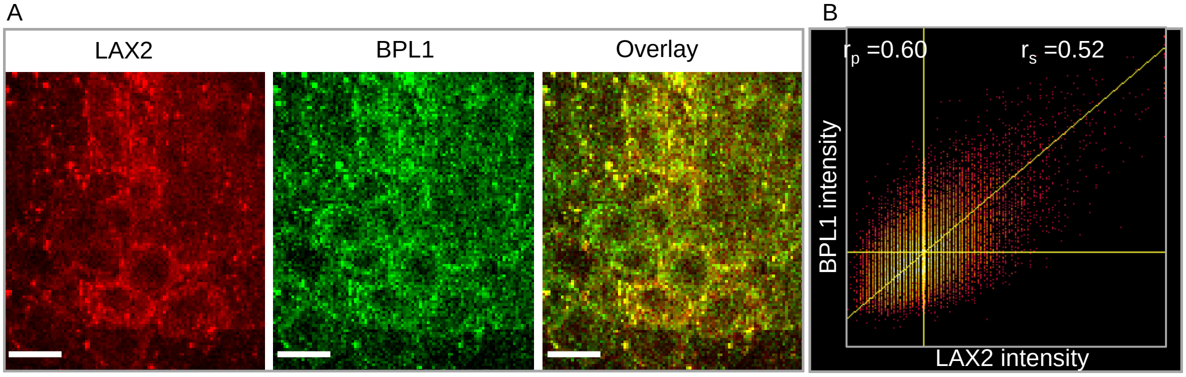

Supplemental Figure S1. LAX2 co-localizes with ER marker BPL1 in *axr4* root cells as revealed by *in situ* immunolocalization. A. Co-localization of LAX2 (red) and ER marker BPL1 (green). B. 2D scatterplot correlation of pixel intensities generated by colocalization threshold ImageJ plugin from a single image. Pearson (rp) and Spearman rank (rs) pixel correlation analysis by Coloc 2 ImageJ plugin, value is the average generated from 3 biological replicates from co-localised whole cells. Scale bar 5 µm.

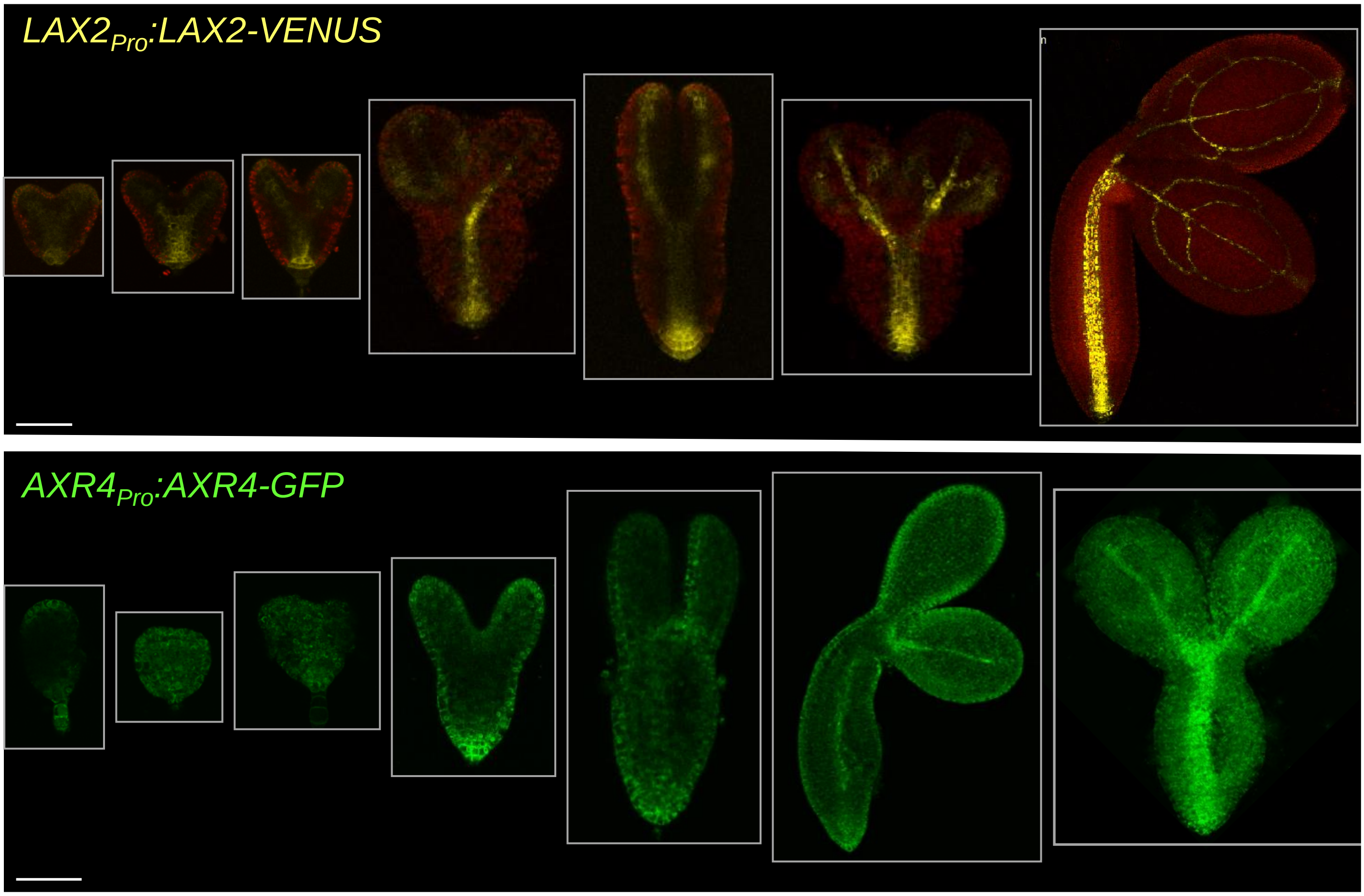

Supplemental Figure S2. *AXR4* is expressed in the expression domains of *LAX2 in the developing embryos.*
Confocal images showing (A) LAX2$_{Pro}$*:LAX2-VENUS and (B) AXR4*$_{Pro}$*:AXR4-GFP expression in various stages of developing embryos.* The last two images in both panels are the same images as in Figure 2 (C,D) but have been included here for comparison. Scale bar 50 µm.

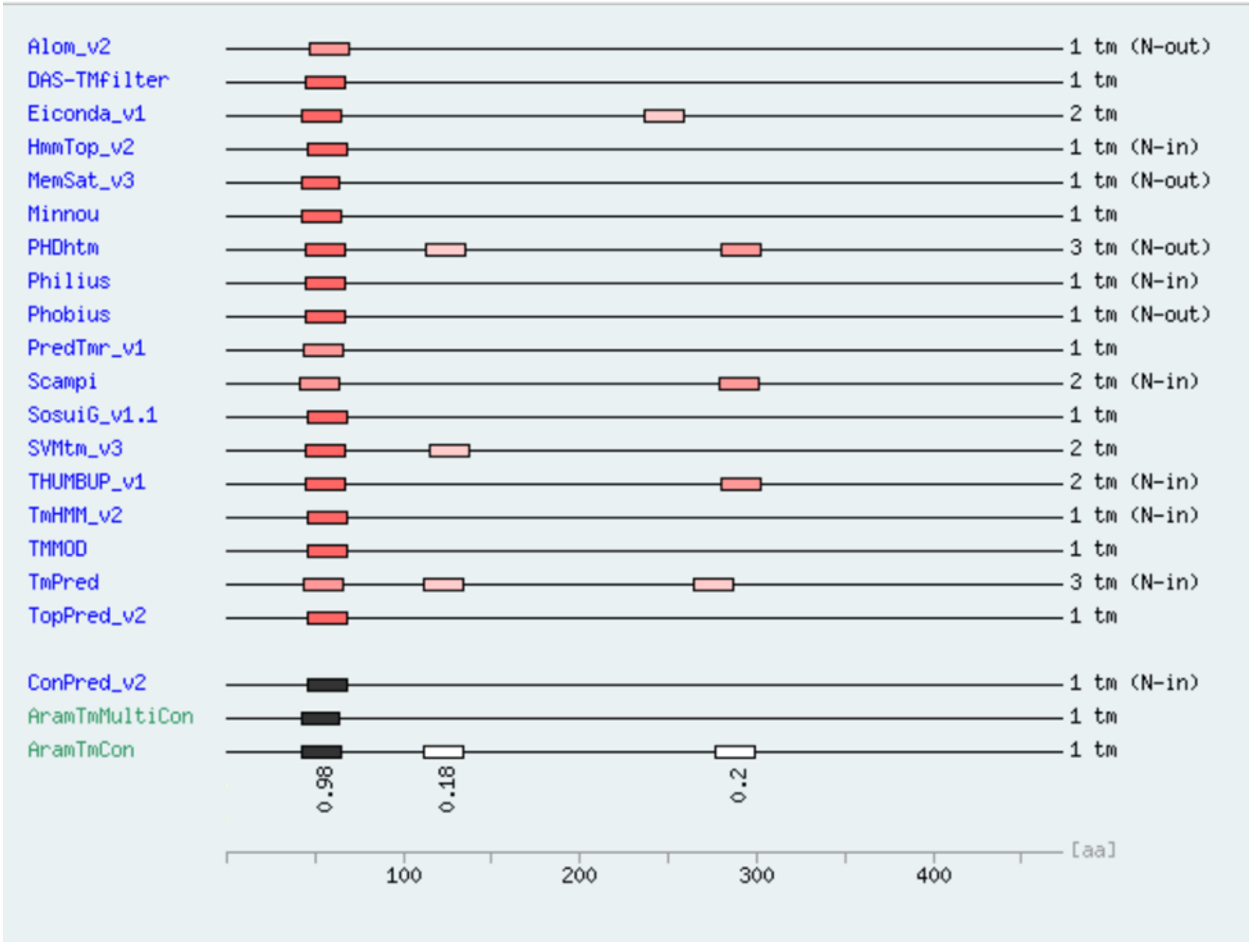

Supplemental Figure S3. Topology Mapping studies strongly suggest that AXR4 has one Transmembrane domain but some studies indicate two or even three TMs.

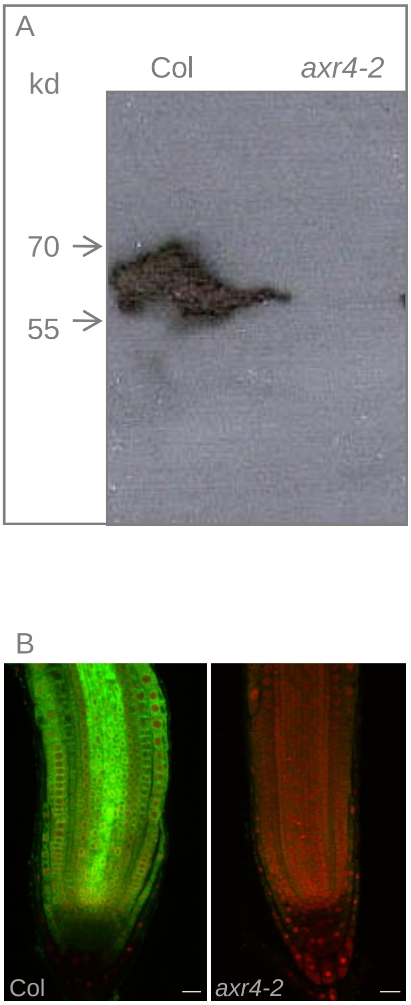

Supplemental Figure S4. AXR4 antibodies are specific for AXR4. Specificity and quality of AXR4 antibodies was tested by Westerns (A) and *in situ* immunolocalization (B). This antibody detects a single correct size band in WT but not in axr4 root extracts (A) and shows a clear localisation in Col roots but not in axr4-2 roots (B) indicating an AXR4 specific antibody. For *in situ* localisation (B), 4-d old roots were treated with anti-AXR4 primary antibody and anti-sheep Alexa Fluor 488 coupled secondary antibody. Background staining was done using Propidium Iodide. Scale bar 10 μm.

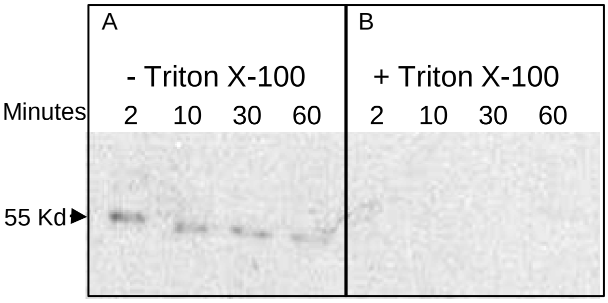

Supplemental Figure S5. Protease protection experiment. Western immuno-detection of AXR4 following proteinsase K treatment of microsomes in absence (A) or presence (B) of triton x 100.

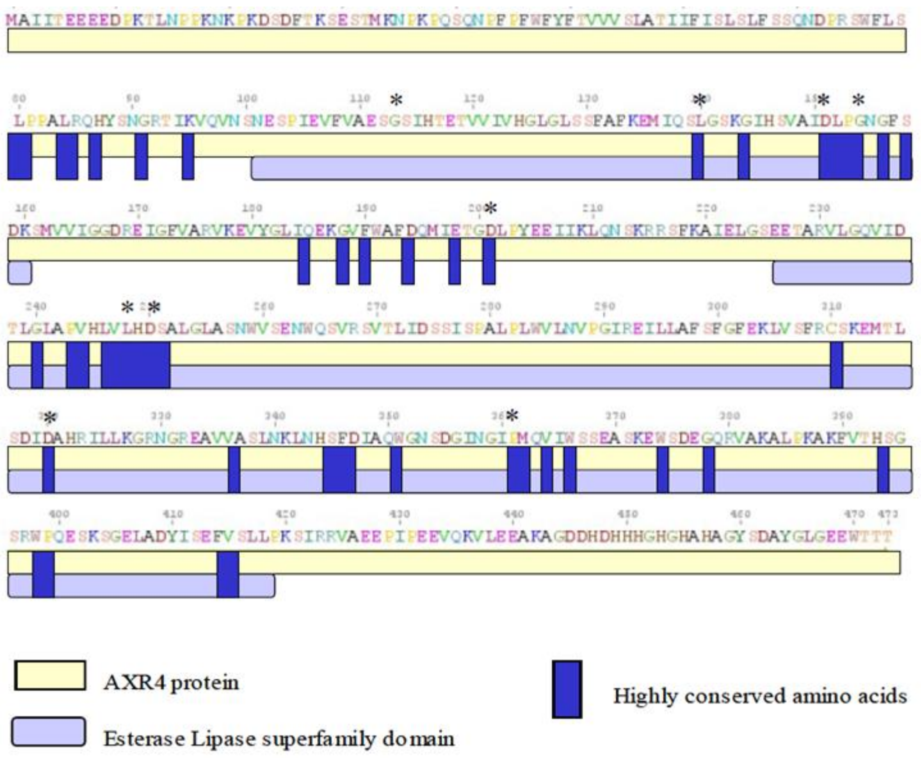

Supplemental Figure S6. Graphic representation of AtAXR4 showing highly conserved residues from the multisequence alignment of AXR4-like sequences. Asterisks represent sites chosen for site directed mutagenesis.

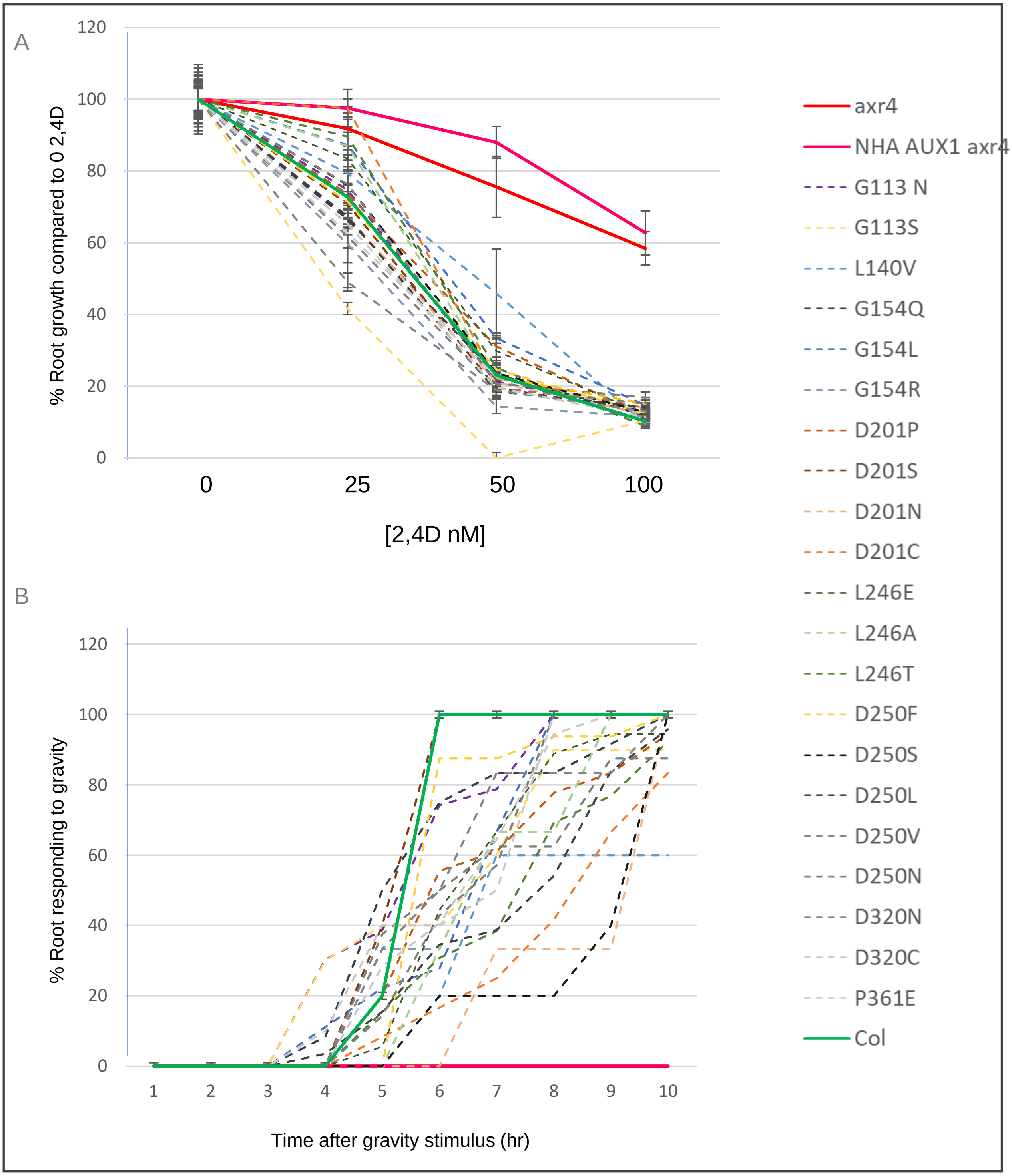

Supplemental Figure S7. Single point mutations in AXR4 are tolerated. Phenotypic analysis of $_{axr4-2}$*mAXR4GFP* lines show restoration of 2,4D sensitivity (A) and root gravitropism (B) defect of axr4-2 mutants. Error bars represent standard error.

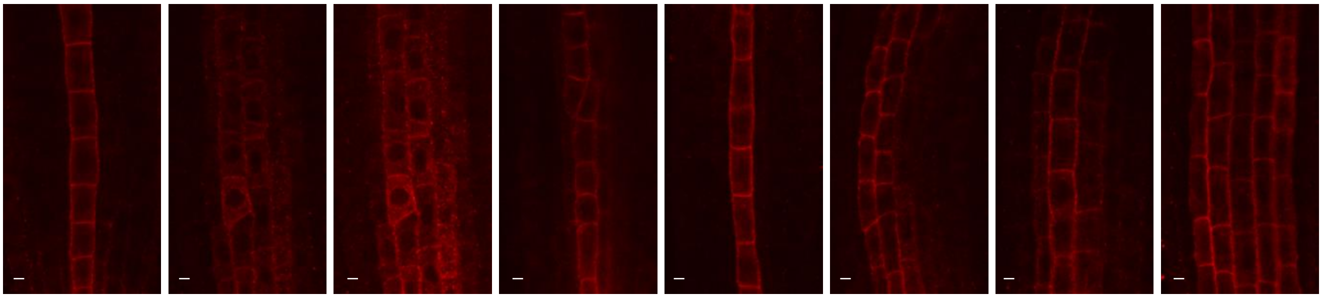

Col | *axr4-2* | *axr4-2* High Gain | G113S | L140V | D201C | L246A | D250V

Supplemental Figure S8. *In situ* immunolocalization of LAX2 in Arabidopsis roots using anti-LAX2 primary antibody and Alexa Fluor 543 coupled secondary antibody in controls (Columbia, *axr4*) and a few IVM lines (G113S, L140V, D201C, L246A and D250V). Scale bar= 2µM.

A

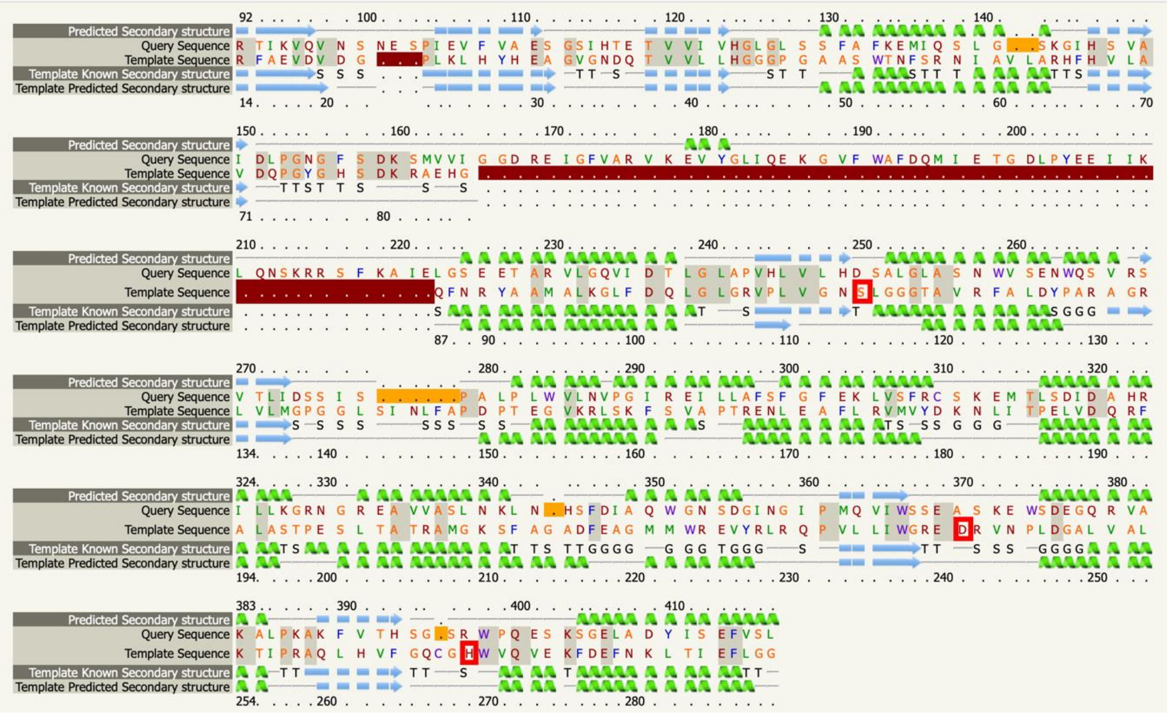

B

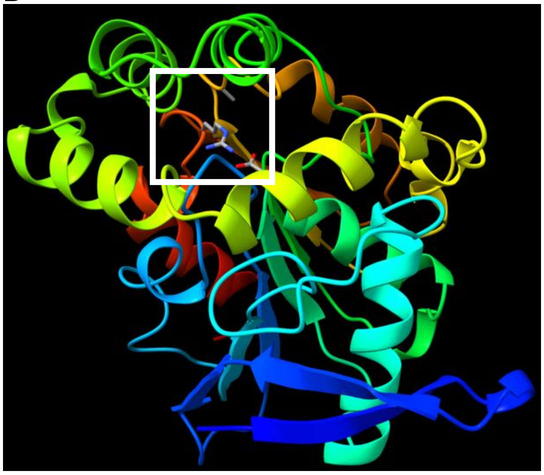

C

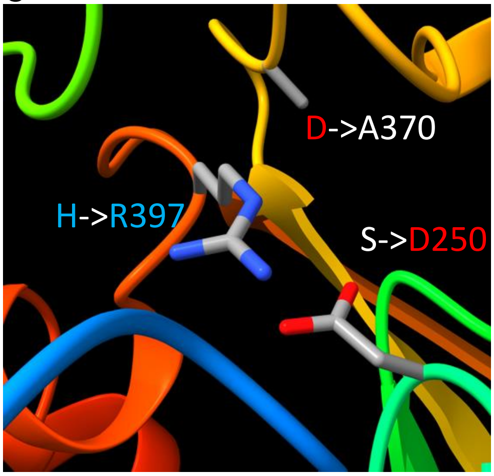

Supplemental Figure S9. Catalytic site is inactive in AXR4. Sequence alignment of AXR4 with 2vf2 whose catalytic residues are outlined by red boxes (A). This sequence was ranked 2nd, but still has 100% confidence, 24% amino acid identity and fewer insertions/deletions than the top-ranked structure. Above and below the alignment are respectively the predicted AXR4 secondary structure and the known and predicted structures for the reference protein. The AXR4 model structure is depicted as a ribbon colour-coded from blue at the N-terminus to red at the C-terminus (B). The white box denotes the location of presumptive active site residues and a close-up view is shown in (C). Deepest in the triad should be an Aspartate (D), but in AXR4 this has become Alanine (A370) so there is not the expected influence on the alkaline residue: usually H but R397 is an acceptable alternative. The position of the nucleophile Serine (S) has been replaced by an Aspartate (D250). Hence, the cavity does not function as a site of hydrolysis.

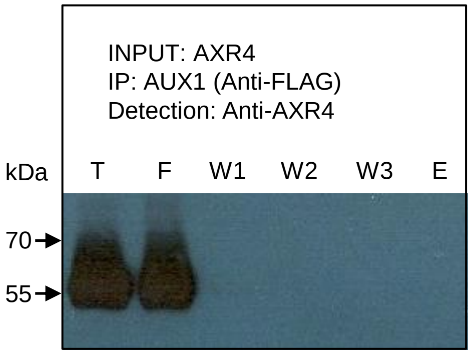

Supplemental Figure S10. Control pull down experiments show that AXR4 can NOT be detected in the pull down with anti-FLAG resin when AXR4 was expressed alone. T: Total extract; F: Flowthrough; W: Wash and E: Elution.

MAIITEEEEDPKTLNPPKNKPKDSDFTKSESTMKNPKPQSQ
NPFPFWFYFTVVVSLATIIFISLSLFSSQNDPRSWFLSLPP
ALRQHYSNGRTIKVQVNSNESPIEVFVAESGSIHTETVVIV
HGLGLSSFAFKEMIQSLGSKGIHSVAIDLPGNGFSDKSMVV
IGGDREIGFVARVKEVYGLIQEKGVFWAFDQMIETGDLPYE
EIIKLQNSKRRSFKAIELGSEETARVLGQVIDTLGLAPVHL
VLHDSALGLASNWVSENWQSVRSVTLIDSSISPALPLWVLN
VPGIREILLAFSFGFEKLVSFRCSKEMTLSDIDAHRILLKG
RNGREAVVASLNKLNHSFDIAQWGNSDGINGIPMQVIWSSE
ASKEWSDEGQRVAKALPKAKFVTHSGSRWPQESKSGELADY
ISEFVSLLPKSIRRVAEEPIPEEVQKVLEEAKAGDDHDHHH
GHGHAHAGYSDAYGLGEEWTTT

Supplemental Figure S11. Motif analysis for AXR4. The N terminal cytosolic domain is underlined. Potential diacidic motif (D/E)X(D/E) are highlighted in blue font. Canonical dilysine motif KNKPK are highlighted in red.

Supplemental Table S1. Primers used for random site directed mutagenesis in the conserved amino acids in the α/β hydrolase fold domain of AXR4

Primer Name	Sequence
AXS 113 F1	GTAGCTGAATCANNTTCGATTCATACAGAGACTG
AXS 113 R1	CTGTATGAATCGAANNTGATTCAGCTACAAAGAC
AXS 140 F1	GAAATGATTCAATCTNNTGGATCAAAAGGGATCCATAG
AXS 140 R1	CCTTTTGATCCANNAGATTGAATCATTTCC
AXS 151 F1	GTGTTGCTATTNNTTTACCTGGAAATGGGTTCTC
AXS 151 R1	CATTTCCAGGTAAANNAATAGCAACACTATGGATC
AXS 154 F1	GCTATTGATTTACCTGGAAATGGGTTCTCTGATAAGTC
AXS 154 R1	GAGAACCCATTTNNAGGTAAATCAATAGCAACAC
AXS 201 F1	GATTGAAACTGGANNTTTGCCTTATGAGGAGATC
AXS 201 R1	CTCATAAGGCAAANNTCCAGTTTCAATCATCTG
AXS 246 F1	GCTCCTGTGCATNNGGTTCTTCATGATTCAGC
AXS 246 R1	CATGAAGAACCNNATGCACAGGAGCTAAACC
AXS 250 F1	GGTTCTTCATNNTTCAGCTTTAGGGTTAGCTTC
AXS 250 R1	CCTAAAGCTGAANNATGAAGAACCAAATGCACAG
AXS 320 F1	CTTTATCGGATATTNNTGCTCATAGGATACTTTTGAAG
AXS 320 R1	GTATCCTATGAGCANNAATATCCGATAAAGTCATC
AXS 361 F1	GGATTAATGGTATTNNGATGCAAGTGATTTGGTCTAG
AXS 361 R1	CACTTGCATCNNAATACCATTAATCCCATCTG
AXS 414 F1	CATATCAGAANNTGTCTCTCTCCTCCCTAAATC
AXS 414 R1	GAGGAGAGAGACANNTTCTGATATGATTACTGCAAG

Supplemental Table S2. Primers used for genotyping of IVM lines

Name	Sequence
GFP5	GACGGGAACTACAAGACACG
GFP6	CCAACTTGTGGCCGAGGATG
